# Assessment of Draxxin^®^ (tulathromycin) as an inhibitor of in vitro growth of *Babesia bovis*, *Babesia bigemina* and *Theileria equi*

**DOI:** 10.1016/j.ijpddr.2018.04.004

**Published:** 2018-04-17

**Authors:** Marta G. Silva, Nicolas F. Villarino, Donald P. Knowles, Carlos E. Suarez

**Affiliations:** a Department of Veterinary Microbiology and Pathology, Washington State University, Pullman, WA, 99164-7040, USA; b Program in Individualized Medicine, Department of Veterinary Clinical Sciences, College of Veterinary Medicine, Washington State University, Pullman, USA; c Animal Disease Research Unit, USDA-ARS, 3003 ADBF, WSU, Pullman, WA, 99163-6630, USA

**Keywords:** Draxxin^®^, Tulathromycin, *Babesia bovis*, *Babesia bigemina*, *Theileria equi*, In vitro inhibition growth

## Abstract

*Babesia bovis, Babesia bigemina* and *Theileria equi* are worldwide tick-borne hemoprotozoan that cause diseases characterized by fever, anemia, weight loss and abortion. A common feature of these diseases are transition from acute to chronic phases, in which parasites may persist in the host for life, and becoming a reservoir for tick transmission. The live-attenuated vaccines for *B. bovis* and *B. bigemina* are not available for worldwide use due to legal restrictions and other concerns such as potential erythrocyte antigen and pathogen contamination, and a vaccine for *T. equi* is not available. The use of chemotherapeutics is essential to treat and control these diseases, but several studies have shown the development of drug-resistance by these parasites, and safe and effective alternative drugs are needed. Tulathromycin, a macrolide antibiotic, has proven to be effective against a vast range of bacteria and *Plasmodium yoelli*, a *Babesia* and *Theileria* related intra-erythrocytic apicomplexan. Draxxin^®^ (tulathromycin) is currently licensed to treat infections that cause respiratory diseases in cattle in several countries. In this study, the activity of Draxxin^®^ was tested in vitro on cultured *B. bovis, B. bigemina* and *T. equi*. Addition of the drug to in vitro cultures resulted in cessation of parasite replication of the three species tested, *B. bovis, B. bigemina* and *T. equi*, with estimated IC_50_ of 16.7 ± 0.6 nM; 6.2 ± 0.2 nM and 2.4 ± 0.1 nM, respectively, at 72 h. Furthermore, neither parasites nor parasite DNA were detectable in cultures treated with IC_100_, suggesting Draxxin^®^ is a highly effective anti-*Babesia*/*Theileria* drug. Importantly, the IC_50_ calculated for Draxxin^®^ for the *Babesia/Theileria* parasites tested is lower that the IC_50_ calculated for some drugs currently in use to control these parasites. Collectively, the data strongly support in vivo testing of Draxxin^®^ for the treatment of bovine babesiosis and equine piroplasmosis.

## Highlights

• Chemotherapeutics are critical to treat bovine babesiosis and equine theileriosis.
• Study of the activity of Draxxin^®^ in vitro *B. bovis, B. bigemina*, *T. equi* cultures.
• Addition of Draxxin^®^ to the cultures resulted in cessation of parasite replication.
• IC_50_ (nM) of 16.7; 6.2 and 2.4 to *B. bovis, B. bigemina* and *T. equi*, respectively.
• Results suggests that Draxxin^®^ is a highly effective anti-*Babesia*/*Theileria* drug.

## Graphical abstract

The effect of different concentrations of Draxxin^®^ (tulathromycin) in in vitro growth of *B. bovis*, *B. bigemina* and *T. equi* parasites at 72 h after drug addition to the culture.

**Figure:**
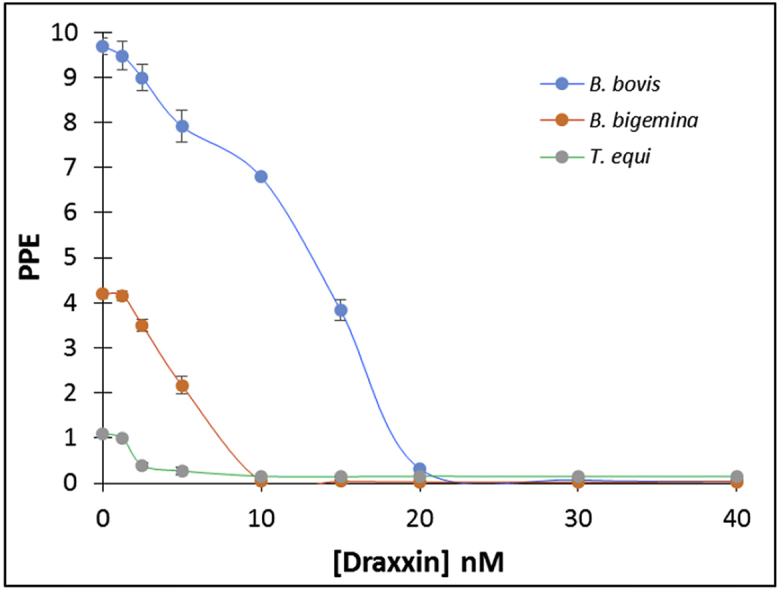

## 1 Introduction

Chemotherapeutics are extremely important for the management of infectious diseases, especially when effective and safe vaccines are unavailable. However, the identification of safe and efficacious parasite-specific chemotherapeutics is time-consuming and expensive. Some of the disadvantages of current chemotherapeutics include increased parasite drug-resistance, the occurrence of drug-associated side-effects, such as toxicity, and the risk of contamination of the food chain (Vial and Gorenflot, 2006; Suarez and Noh, 2011). Bovine and equine piroplasmosis are tick-borne diseases present worldwide, causing significant economic losses due to decreases in meat and milk production, abortion, anemia and high mortality rate of susceptible animals in endemic areas (Uilenberg, 1995). The tick-borne apicomplexan parasites *Babesia bovis* and *Babesia bigemina* are the most prevalent *Babesia* species that infect cattle worldwide. The related tick-borne apicomplexan *Theileria equi* has significant economic consequences, especially in equine sport activities and the racing industry through restricting movement of infected horses (Wise et al., 2013). Importantly, both, cattle and horses surviving acute infections become persistent reservoirs allowing parasite transmission by ticks. Currently, there are live-attenuated vaccines available for the prevention of acute bovine babesiosis, but not for *T. equi*. However, these vaccines have severe limitations, such as the inability to protect against infection and are not licensed for use in all countries, including the United States of America. Available chemotherapeutics for bovine and equine piroplasmosis include imidocarb dipropionate (Imizol^®^, Schering-Plough Animal Health) and diminazene aceturate (Berenil^®^, Intervet, India Pvt. Ltd.). However, imidocarb dipropionate may have negative secondary effects in treated animals, and may result in salivation, vomiting, diarrhea and injection site inflammation (http://www.merck-animal-health-usa.com/products/130_163327/productdetails_130_163631.aspx#/product/canine/Imizol/1). Furthermore, long term use of these treatments invariably results in the emergence of drug-resistant parasites (Mosqueda et al., 2012; Hines et al., 2015).

Therefore, taking these considerations together, the diseases caused by these apicomplexan parasites may prove to become intractable, and new anti-parasitic drugs are urgently needed. Tulathromycin is a semi-synthetic macrolide antibiotic of the subclass triamilide (https://www.zoetisus.com/products/pages/draxxin_index/index.aspx). Importantly, a specific formulation (currently commercialized as Draxxin^®^) of this drug has been already approved for the treatment of respiratory disease, keratoconjunctivitis and foot rot in cattle in several countries including the US. Therefore, this drug is currently approved by the FDA and widely commercialized, greatly facilitating the process for new potential applications. A previous study demonstrated that tulathromycin has antimalarial activity in in vivo mice infected with *Plasmodium yoelli* (Villarino et al., 2015). Based on these preliminary observations and in the fact that *Plasmodium*, *Theileria* and *Babesia* are related intra-erythrocytic apicomplexan parasites, we hypothesized that Draxxin^®^ (tulathromycin) inhibits the development of *Babesia* and *Theileria* parasites in in vitro cultures. The study presented here evaluates the use of a commercial available chemotherapeutic, Draxxin^®^ (tulathromycin) as a drug that inhibits the in vitro growth of *B*. *bovis, B*. *bigemina* and *T*. *equi*.

## 2 Material and methods

### 2.1 In vitro cultivation of the B. bovis, B. bigemina and T. equi

*Babesia bovis* Texas strain, *B*. *bigemina* Puerto Rico strain and *T*. *equi* Florida strain cultures were grown in long-term microaerophilous stationary-phase as described by Levy and Ristic (1980), Vegas et al. (Vega et al., 1985) and Zweygarth et al. (1995), respectively. Briefly, parasites were grown in a supplemented HL-1 medium at pH 7.2, with 10% and 5% hematocrit of bovine erythrocytes (*B*. *bovis* and *B*. *bigemina*, respectively) or with 10% hematocrit equine erythrocytes (*T*. *equi*). Cultures were maintained at 37 °C in an atmosphere of 5% CO_2_, 5% O_2_ and 90% N_2_ and media was replaced daily.

### 2.2 In vitro growth inhibition assay

In vitro inhibition assays for *B. bovis, B. bigemina* and *T. equi* parasites were performed to evaluate the effect of the Draxxin^®^ (tulathromycin). This study was performed in 96-well plates using 180 μl cultures per well containing the different Draxxin^®^ concentration tested (1.56 nM, 3.1 nM; 6.2 nM; 12.4 nM; 18.6 nM; 24.8 nm; 37.2 nM and 49.6 nM) diluted in culture medium, with an initial percentage of parasitized erythrocytes (PPE) of 0.2% in all subcultures. The first screening was to identify if Draxxin^®^ has an inhibitory effect on *Babesia* spp. and *T. equi* strains and calculate the 50% inhibitory concentrations (IC_50_). Cultures were grown in the presence and absence of Draxxin^®^, and non-infected erythrocytes were also kept in culture media as baseline data for flow cytometry analysis. The culture medium was replaced daily with 150 μl medium per well containing the respective Draxxin^®^ concentration. Cultures were also monitored daily up to 72hr by flow cytometry to measure the PPE and by Giemsa-stained slides for any morphological changes.

A new set of in vitro growth inhibition assays was performed targeting a starting PPE of 1% for *B. bovis* and *B. bigemina* and 9% for *T. equi*. This assay was performed using of Draxxin^®^ at IC_100_: *B. bovis* (37.2 nM), *B. bigemina* (12.4 nM) and *T. equi* (18.6 nM). Cultures grown in the absence of Draxxin^®^ were used as control for no parasite growth inhibition, and culture of non-infected erythrocytes maintained in medium was used as negative control. The culture medium was replaced daily with 150 μl medium per well containing the respective Draxxin^®^ concentration for a period of 72 h. After 72 h, parasites were cultivated in media (in the absence of Draxxin^®^), with splits every 48 h for a period of 8 days. The PPE was evaluated at 72 h and 8 days by flow cytometry.

The effect of the Imizol^®^ (imidocarb dipropionate) was used as a positive control for the in vitro inhibition assays for *B. bovis*, *B. bigemina* and *T. equi* parasites, using an identical protocol as described above. The drug concentrations used were: 1 μM for *B. bovis* and for *T. equi*, and a set of different concentrations (0.001 nM, 0.01 nM; 0.1 nM; 1 nM; 10 nM; 1 μM) for the calculation of the IC_50_ of Imizol^®^ for *B. bigemina*. The initial PPE used was 0.2% in all subcultures. Cultures were monitored daily at 72 h by flow cytometry to measure the PPE.

All experiments were carried out in triplicate for each concentration tested. The IC_50_ values were calculated using GraphPad Prism 7 software by fitting the PPE values (wells treated with Draxxin^®^ at different concentration compared to the positive control well) in a nonlinear regression with a confidence interval of 95% (95% CI). Total inhibitory concentrations (IC_100_) was calculated as the doses of drug required to inhibit the parasite growth to an identical level as found for non-infected erythrocytes (approximately 0.1%).

### 2.3 Flow cytometric method for detection of parasite growth

Flow cytometric assay was performed as described by Wyatt et al. (1991). Briefly, cultures were centrifuged at 450 × *g* for 5 min at 4 °C. The supernatant was discarded, the cell pellet was washed twice with 150 μl of phosphate buffer saline (PBS) pH 7.2 and then, the cell pellet was resuspended in 200 μl of 25 μg/μl Hydroethidine (HE) (Invitrogen) and incubated in 5% CO_2_ incubator at 37 °C for 20 min in the dark. After incubation, excess of HE was washed from the cells by the addition of 200 μl of PBS followed by centrifugation at 450 × *g* for 5 min at 4 °C. The supernatant was discarded and the cell pellet was resuspended in 200 μl of fresh PBS. Then, resuspended cells were transferred to samples tube containing approximately 3 ml of PBS containing 0.2% sodium azide for analysis by flow cytometry using a FACSCaliber (Becton Dickinson) at a flow rate of approximately 2500 events/s with 50,000 events collected. Data was analyzed by FCS Express 3 software (De Novo Software). Stained non-infected erythrocytes were used as a negative control and gated to determine the percentage between infected and non-infected erythrocytes.

### 2.4 Quantitative real-time PCR

Quantitative real-time PCR was performed to assess the copy numbers of *B. bovis merozoite surface antigen* (*msa*)-1 gene, *B. bigemina limulus coagulation factor C domain protein* (*ccp*)-3 and *T. equi erythrocyte merozoite antigen* (*ema*)-1 genes in parasites treated with IC_50_ and IC_100_ concentrations and non-treated parasites (control well) at 72 h post-addition of Draxxin^®^. For *B. bovis* the IC_50_ and IC_100_ concentrations are 16.7 nM and 37.2 nM, respectively; for *B. bigemina* the IC_50_ and IC_100_ concentrations are 6.2 nM and 12.4 nM, respectively; and for *T. equi* the IC_50_ and IC_100_ concentrations are 2.4 nM and 18.6 nM, respectively. The set of primers, PCR cycling and reaction were performed as described by Bastos et al. and Silva et al. (Bastos et al., 2013; Silva et al., 2016). Five μl of plasmid DNA (pDNA) was used for the standard curve and two μl of gDNA was used as samples. Data was analyzed by CFX Manager™ Software (Bio-Rad). Copy numbers of *B. bovis msa-1*, *B. bigemina ccp-3* and *T. equi ema*-*1* genes were calculated based on a standard curve (Bastos et al., 2013).

### 2.5 Statistical analysis

Statistical significance was determined using a two-sample *t*-test for differences among the treatments and one-way parametric ANOVA (GraphPad Prism 7 software). *P* < 0.05 were considered statistically significant. The area under the concentration (AUC) values was calculated using GraphPad Prism 7 software.

## 3 Results

### 3.1 The in vitro inhibitory effect of Draxxin^®^ in growth of B. bovis, B. bigemina and T. equi parasites

**Fig. 1:**
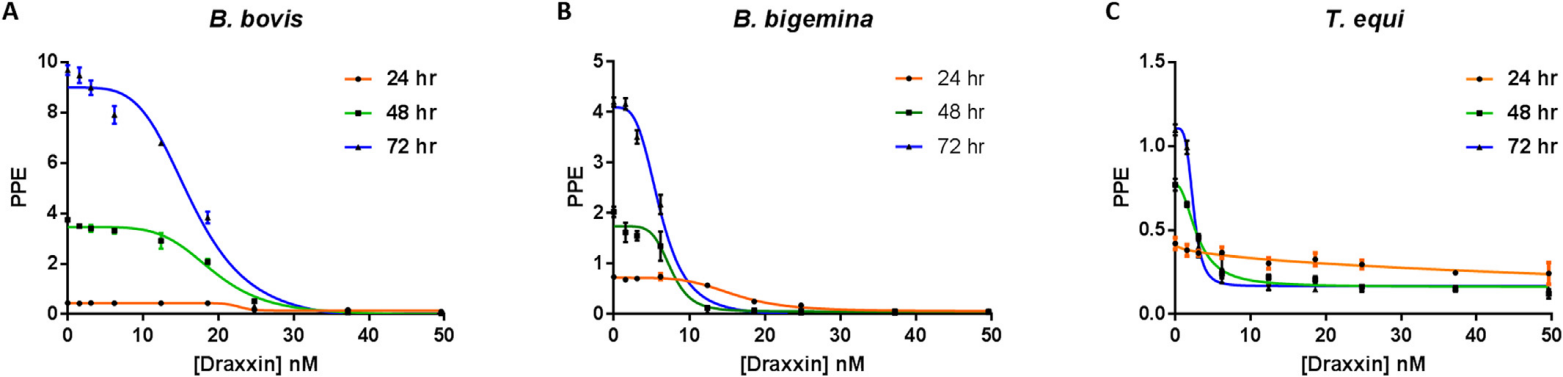
Growth curve obtained at 24 h (orange line), 48 h (green line) and 72 h (blue line) after the addition of different concentrations of Draxxin^®^ in: A) *B*. *bovis*, B) *B. bigemina* and C) *T*. *equi* in in vitro culture. Parasites grown without addition of Draxxin^®^ are represented as “0”. Assay was carried out in triplicate. Error bars indicate standard error of the means for each Draxxin^®^ concentration tested. (For interpretation of the references to colour in this figure legend, the reader is referred to the Web version of this article.)

We initially tested the efficacy of Draxxin^®^ to inhibit the in vitro growth of *B. bovis, B. bigemina and T. equi* parasites. The in vitro growth of those parasites was inversely related to the concentrations of Draxxin^®^ applied, until no growth of parasites was reached (Fig. 1A–C). In addition, daily measurements of the PPE over a 72 h lapse, indicates that the drug effect is also dependent on time, with lower PPE occurring upon longer exposures to the drug (Fig. 1A–C).

The IC_50_ for each parasite tested were determined at 72 h after the addition of the drugs (Table 1). Overall, the growth inhibition data suggests that *T. equi* is the most “susceptible“ of the three parasites tested to the effect of Draxxin^®^, with a calculated IC_50_ of 2.4 nM; followed by *B. bigemina* (IC_50_ of 6.2 nM), and *B. bovis* (IC_50_ of 16.7 nM). In addition, the calculated IC_100_ values were 37.2 nM, 12.4 nM, and 18.6 nM for *B. bovis, B. bigemina and T. equi* parasites, respectively (Table 1).

**Table 1:** IC_50_ and IC_100_ values obtained on *B*. *bovis*, *B*. *bigemina* and *T*. *equi* at 24 h, 48 h and 72 h after treatment with Draxxin^®^.

Species	IC_50_ (nM)			IC_100_ (nM)		
	24 h	48 h	72 h	24 h	48 h	72 h
*B. bovis*	22.8 ± 2.0 N.A.	19.3 ± 0.5 (18.2–20.3)^a^	16.7 ± 0.6 (15.4–17.9)^a^	49.6	37.2	37.2

*B. bigemina*	15.9 ± 0.5 (15–17.1)^a^	7.6 ± 0.6 (7.2–8.9)^a^	6.2 ± 0.2 (5.8–6.6)^a^	37.2	18.6	12.4


*T. equi*	N.A. N.A.	2.9 ± 0.2 (2.6–3.3)^a^	2.4 ± 0.1 (2.2–2.6)^a^	N.A.	24.8	18.6

a 95% CI.

**Suppl. Fig. 1:**
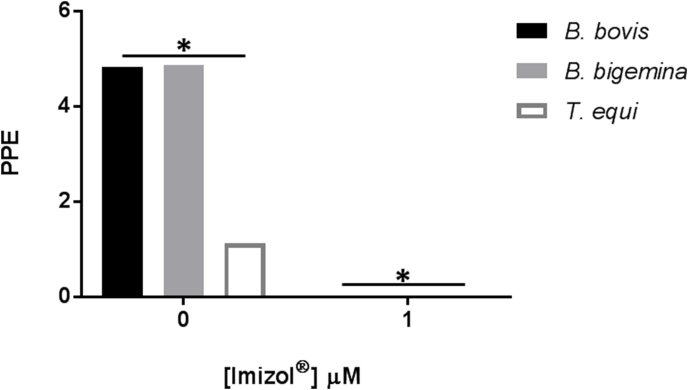
Growth curve of parasites obtained at 72 h after the addition of Imizol^®^ at 1 μM in in vitro culture. Parasites grown without addition of Imizol^®^ are represented as “0”. Assay was carried out in triplicate. Error bars indicate standard error of the means for each sample tested. (*) Represents *p*-value <0.05 indicating a statistically significant difference between media and Imizol^®^ using Student's t-test.

**Suppl. Fig. 2:**
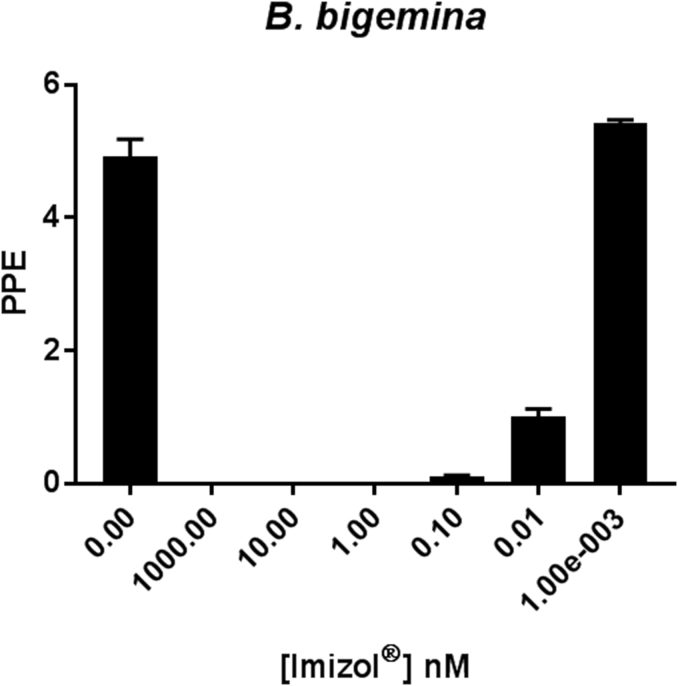
Growth curve obtained at 72 h after the addition of different concentrations of Imizol^®^ in *B. bigemina* in in vitro culture. Parasites grown without addition of Imizol^®^ are represented as “0”. Assay was carried out in triplicate. Error bars indicate standard error of the means for each sample tested.

The Imizol^®^ was able to completely inhibit the in vitro growth of *B. bovis, B. bigemina and T. equi* (Suppl. Fig. 1). We decided to estimate the Imizol^®^ IC_50_ for *B. bigemina* (Suppl. Fig. 2 and Table 2) since, at least to our knowledge, there is no in vitro data for *B. bigemina* parasites. Table 2 also compares the IC_50_ values for the three currently used anti *Babesia/Theileria* drugs with Draxxin^®^.

**Fig. 2:**
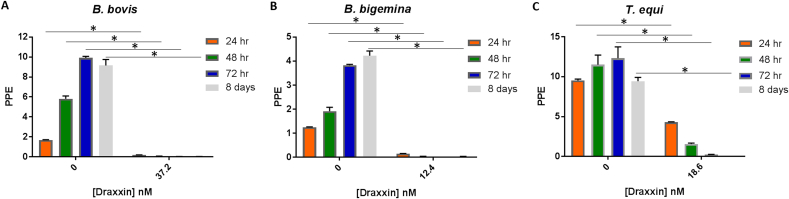
Growth curve of parasites obtained at 24 h (orange bars), 48 h (green bars) and 72 h (blue bars) after the addition of respective IC_100_ of Draxxin^®^, and 8 days (grey bars) without addition of Draxxin^®^ in: A) *B*. *bovis* (37.2 nM), B) *B. bigemina* (12.4 nM) and C) *T*. *equi* (18.6 nM) in in vitro culture. PPE values are in y-axis and Draxxin^®^ concentrations tested are in x-axis. Parasites grown without addition of Draxxin^®^ are represented as “0”. Assay was carried out in triplicate. Error bars indicate standard error of the means for each Draxxin^®^ concentration tested. (*) Represents *p*-value <0.05 indicating a statistically significant difference between media and correspondent Draxxin^®^ using Student's t-test. (For interpretation of the references to colour in this figure legend, the reader is referred to the Web version of this article.)

**Table 2:** IC_50_ values obtained on *B*. *bovis*, *B*. *bigemina* and *T*. *equi* at 72 h after treatment with diminazene aceturate, imidocarb dipropionate, clofazimine and Draxxin^®^.

Species	IC_50_ (nM)		
	*B. bovis*	*B. bigemina*	*T. equi*
Diminazene aceturate (Rizk et al., 2015)	400 ± 190	200 ± 160	770 ± 280
Imidocarb dipropionate *(Nott et al., 1990); ^$^(Rodriguez and Trees, 1996); ^#^(Gopalakrishnan et al., 2016); ^@^in this study	8.6*^$^	0.08^@^	279^#^
Clofazimine (Tuvshintulga et al., 2016)	4500 ± 300	3000 ± 200	290 ± 30
**Draxxin**^**®**^	**16.7 ± 0.6** (15.4–17.9)^a^	**6.2 ± 0.2** (5.8–6.6)^a^	**2.4 ± 0.1** (2.2–2.6)^a^

a 95% CI.

### 3.2 Effects of the application of Draxxin^®^ at a IC_100_ doses into cultures with distinct B. bovis, B. bigemina and T. equi parasitemia levels

The performance of the drug might be affected by the existing parasite load in a given infected animal. The effects of the addition of the calculated IC_100_, into in vitro blood stage cultures with different starting PPE (0.2 and 1% for both *Babesia* parasites and 0.2 and 9% for *T. equi*) were also tested.

**Fig. 3:**
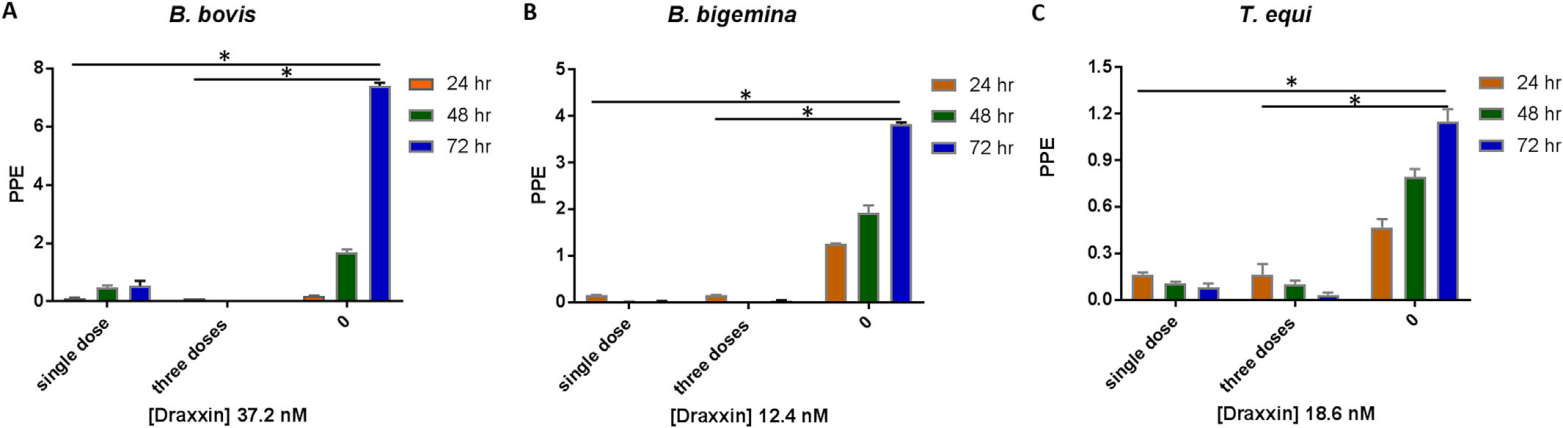
Growth curve of parasites obtained at 24 h (orange bars), 48 h (green bars) and 72 h (blue bars) after the addition of respective IC_100_ of Draxxin^®^ on a single dose (0 h) or three doses (over 72 h) in: A) *B*. *bovis* (37.2 nM), B) *B. bigemina* (12.4 nM) and C) *T*. *equi* (18.6 nM) in in vitro culture. PPE values are in y-axis and Draxxin^®^ concentrations tested are in x-axis. Parasites grown without addition of Draxxin^®^ are represented as “0”. Assay was carried out in triplicate. Error bars indicate standard error of the means for each Draxxin^®^ concentration tested. (*) Represents *p*-value <0.05 indicating a statistically significant difference between media and correspondent Draxxin^®^ using Student's t-test. (For interpretation of the references to colour in this figure legend, the reader is referred to the Web version of this article.)

Parasites were not able to growth in the cultures with the addition of 37.2 nM, 12.4 nM and 18.6 nM of Draxxin^®^ in *B. bovis, B. bigemina* and *T. equi* cultures, respectively (Fig. 2A–C) regardless of their initial PPE. In addition, no parasites were detected at 8 days after treatment with these doses (*P* < 0.05) (Fig. 2A–C), suggesting the lack of Draxxin^®^-resistant parasites. An additional follow up study was performed where cultures were treated with a single IC_100_ dose (time 0). No parasite growth was evident in the following 72 h, which was the full duration of the study (Fig. 3A–C). In case of *T. equi* parasites, the lack of growth was observed at 72 h after treatment (*P* < 0.05) but the parasite growth was significantly decreased at 24 h and 48 h after treatment (*P* < 0.05).

The AUC estimated values for *B. bovis, B. bigemina* and *T. equi* cultures at IC_100_ values and after 72 h with Draxxin^®^ was 720 ng*hr/μl, 1680 ng*hr/μl and 1080 ng*hr/μl, respectively.

### 3.3 Quantitation of parasites using qPCR and calculation of IC_50_ and IC_100_ concentration of Draxxin^®^ for B. bovis, B. bigemina and T. equi

**Fig. 4:**
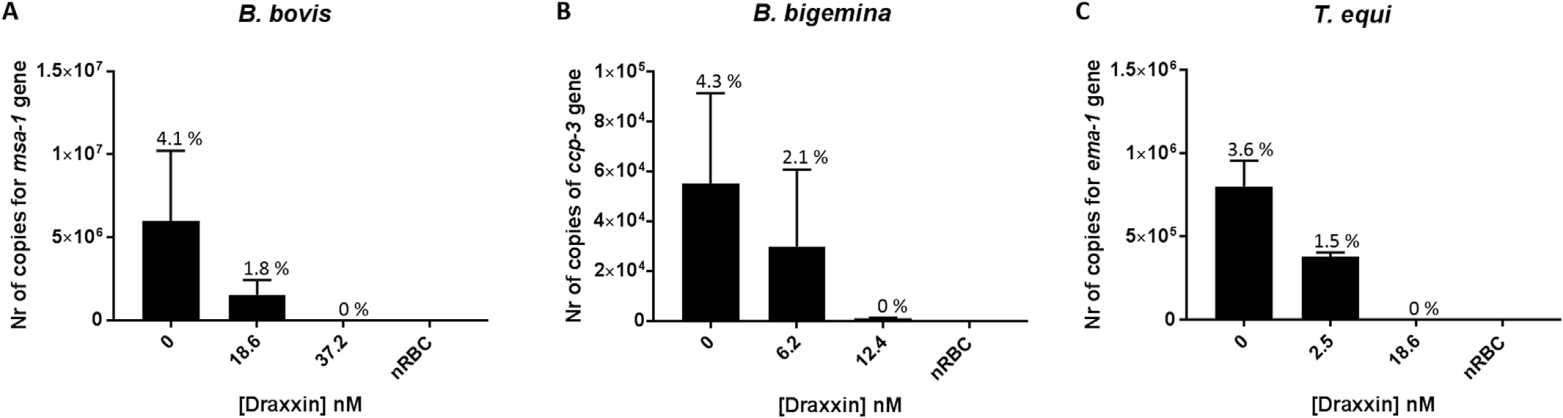
Number of copies obtained from the quantitative PCR at IC_50_ and IC_100_ and non-treated control well (parasites grown without the addition of Draxxin^®^, represented as “0”). A) *B*. *bovis*, B) *B. bigemina* and C) *T*. *equi*. On the top of each bar is presented the PPE quantified. Assay was carried out in triplicate, and samples analyzed individually after 72 h in culture. Error bars indicate the standard error of the means for each sample analyzed. (*) Represents *p*-value <0.05 indicating a statistically significant difference between media and correspondent Draxxin^®^ using one-way ANOVA.

Cultured parasites were treated with either IC_50_ and IC_100_ doses of Draxxin^®^ for 72 h. Total gDNA was extracted from all cultures at 72 h, and analyzed by qPCR. While quantitative PCR detected parasite DNA in all samples treated with IC_50_, no gDNA was detected from the samples collected from cultures treated with IC_100_ doses for *B. bovis* and *T. equi*. However although qPCR detected 0.17% of total parasites in *B. bigemina* gDNA samples derived from parasites treated with IC_100,_ no parasites were detected by FACS. The PPE of *B. bovis*, *B. bigemina* and *T. equi* cultures in the absence of Draxxin^®^, IC_50_ and IC_100_, respectively, are shown in Fig. 4-C. The results are consistent with full abrogation of parasite growth at 72 h, when the cultures are treated with an IC_100_ for the three parasites tested.

## 4 Discussion

Treatment of acute bovine babesiosis and equine theileriosis is achieved by using chemotherapeutics, for example, imidocarb dipropionate. However, studies have shown emerging resistance to imidocarb dipropionate by these parasites which leads to the need of new, safe and effective drugs (Mosqueda et al., 2012; Hines et al., 2015). This study demonstrates inhibition of the in vitro growth of *B. bovis, B. bigemina and T. equi* parasites by the commercially available antibiotic Draxxin^®^ at relatively low concentrations (nM). Interestingly, *B. bovis* parasites require 3 times more Draxxin^®^ to fully inhibit the parasite growth, with an IC_50_ which is approximately 3 times higher than the IC_50_ of *B. bigemina*, and approximately 7 times higher than the IC_50_ of *T. equi*. The reasons for increased IC_50_ in *B. bovis* remain unknown, but could be related to the mechanism(s) of drug action. It was shown that macrolide antibiotics binds with the 23S prokaryotic rRNA (Schlunzen et al., 2001; Hansen et al., 2002; Andersen et al., 2012). It is likely that the antibiotic interferes with the protein synthesis machinery of the plastid organelle present in most apicomplexan parasites. This organelle is the result of an ancient symbiotic association of the ancestor of apicomplexans with a red algae containing a primary photosynthetic endosymbiont, and its function is essential for the survival of the parasites, thus is a possible drug target (Wiesner et al., 2008). It is possible that the differences in the Draxxin^®^ IC_50_ values among the distinct parasites may be related to sequence variation occurring in the targeted 23S rRNA present in the plastid organelles (Hansen et al., 2002; Wiesner et al., 2008). Or alternatively, to other undefined pathways related to the mechanisms of action that can be due to a single factor or a combination of several factors. These include a distinct rate of degradation or elimination of the drug by the parasites, the accessibility of the drug to the actual target molecule(s), interaction of the drug with distinct competing molecules in the parasites, *etc*.

In this study, we addressed the possible existence of Draxxin^®^ resistant parasites by testing for parasite DNA in long term cultures treated with IC_100_ concentrations of the drug for 8 days. Flow cytometric analysis performed on the 8th day and negligible amounts of parasite DNA as detected by quantitative PCR supports the conclusion that no parasites survived Draxxin^®^ treatment.

In addition, application of IC_100_ was able to completely abrogate the growth of the three parasite species tested, independent of the starting PPE. Most persistently infected animals in the field maintain PPE levels that are undetectable by light microscopy. However, acute infections, and outbreaks caused by highly virulent strains, or infections affecting immunocompromised animals, may result in much elevated parasite loads. Having a drug that is effective against high PPEs of acute infections is critical.

Importantly, in all three species tested, the in vitro IC_50_ calculated for Draxxin^®^ is at least six fold lower than the values calculated for diminazene aceturate (Rizk et al., 2015) which is currently approved for use in cattle and equine treatment of babesiosis/piroplasmosis. In addition, the IC_50_ calculated for Draxxin^®^ is significantly lower (100 fold) than the IC_50_ calculated for clofazimine, a drug that demonstrated inhibitory effect in in vitro, but remains unlicensed for in vivo use (Tuvshintulga et al., 2016).

In addition, the IC_50_ value for *T. equi* for Draxxin^®^ is one fold lower than the IC_50_ values for imidocarb dipropionate (Gopalakrishnan et al., 2016). Also, the imidocarb dipropionate IC_50_ value calculated for *B. bigemina* using an in vitro assay and reported for the first time in this study, is lower than the value calculated for *B. bovis*. The *B. bovis* IC_50_ for Draxxin^®^ is two times higher than the value previously calculated for imidocarb dipropionate (Nott et al., 1990; Rodriguez and Trees, 1996). Together, these data suggest that the active principle of Draxxin^®^ might be considered a reasonable option for treatment of cattle and horses infected with *Babesia* and *Theileria* parasites, respectively, especially since resistance to imidocarb dipropionate in *T. equi* has been reported recently (Hines et al., 2015). However effective and safe dosage regimens remains to be established.

Draxxin^®^ is approved by Food and Drug Administration and the European Medicine Agency for use in veterinary species. The antibacterial effect has been well-known and study in beef and dairy calves, and in swine. The in vitro data estimated for the AUC versus time curve of Draxxin^®^ in our study, was comparable to the AUC reported for cattle treated with the FDA-approved label dose (2.5 mg/kg) for treating pneumonia (Nowakowski et al., 2004). This suggests that the currently approved dose can be also be applicable for the treatment of babesiosis and theileriosis in large animals.

To our knowledge, this study showed for the first time the inhibitory effects of Draxxin^®^ in *Babesia* spp. and *T. equi* parasites. The data demonstrates the potential for the development of Draxxin^®^ as a novel pharmacological intervention against *Babesia*/*Theileria* spp. parasites. Furthermore, the results of this study permit us to speculate that other commercially available macrolides for treating pneumonia in cattle (gamithromycin, tilosin and tilmicosin) would also be active against *B. bovis*, *B. bigemina* and *T. equi*.

In conclusion, Draxxin^®^ showed a full inhibitory effect on the in vitro growth of *B. bovis*, *B. bigemina* and *T. equi*. Draxxin^®^ is commercially available currently, and approved for use in beef and dairy cattle. Future studies will evaluate the in vivo efficacy of the FDA-approved dosage regimen for the treatment of *B. bovis*, *B. bigemina* infection in cattle and *T. equi* in horses. The process for its worldwide approval for the treatment of these parasitic diseases can be highly facilitated, considering that the human, food and animal safety of the FDA-approved dosage regimen is well established.

## Authors’ contributions

MGS, NV and CES designed the study. MGS performed the in vitro studies and statistical analysis. MGS, NV, DK and CES wrote the manuscript. All authors read and approved the final version of the manuscript.

## Conflicts of interest

The authors declare no competing interests.
